# Chronic renal disease detection developments with future potential to improve veterinary clinical practice

**DOI:** 10.3389/fvets.2026.1874059

**Published:** 2026-09-23

**Authors:** M. Arevalo Turrubiarte, H. Rideout, A. D. Whetton, N. Geifman, S. K. Armstrong

**Affiliations:** 1 Faculty of Health and Medical Sciences, School of Veterinary Medicine, Veterinary Health Engine (vHive), University of Surrey, Guildford, United Kingdom; 2 Faculty of Health and Medical Sciences, School of Biosciences, Veterinary Health Engine (vHive), University of Surrey, Guildford, United Kingdom; 3 Faculty of Health and Medical Sciences, School of Health Sciences, Veterinary Health Engine (vHive), University of Surrey, Guildford, United Kingdom

**Keywords:** biomarkers, canine, diagnostic, feline, human, kidney, veterinary

## Abstract

Chronic Kidney Disease (CKD) is a major global human and veterinary health concern. Novel blood and urine biomarkers currently offer significant opportunities to advance early diagnosis, prognosis, and clinical outcomes. Adopting a translational medicine approach, we summarise research performed to date on CKD biomarkers in humans and relate these findings to companion animal CKD biomarker research. Emerging candidates, including microRNAs and multiprotein measurements for diagnostic signatures, are also considered. We discuss the potential complementarity offered by multi-omic approaches and Artificial Intelligence (AI) driven diagnostic algorithm generation for the understanding of CKD pathogenesis in dogs and cats.

## 1 Introduction

Chronic Kidney Disease (CKD) affects companion animals, although its true prevalence within these populations remains uncertain. In dogs, estimated rates range from 0.5 to 10%, whereas in cats, prevalence is estimated to be between 1.0–3.0% over their lifetime (1, 2). However, within the geriatric cat population the prevalence increases markedly to 30–80% (3). This wide variation highlights the need for more robust, standardised prevalence studies. A study has investigated the impact of risk factors on survival time in dogs; however, other variables, including the diagnostic methods used, age, and CKD stage, should also be considered. Furthermore, findings from a specific study population may not be representative of the wider canine population, particularly when the breeds included are similar (4).

Various factors are reported to predispose and increase risk of CKD in companion animals. For example, certain breeds have an increased CKD risk such as the Shar Pei, Bull Terrier, West Highland Terrier and Boxer. In cats breeds more commonly affected are Abyssinian, Ragdoll, Burmese, and Maine Coon (4, 5). Age and sex are also factors that increase CKD likelihood with geriatric pets far more frequently diagnosed (1). There is also evidence to suggest, neutered males are more likely to develop clinical signs earlier than neutered females (6). Diet is another contributing factor, particularly in cats. Diets high in protein, associated potassium depletion, with or without increased phosphorus content, are linked to the development of CKD (7). Generally, dogs affected by CKD have a worse prognosis than affected cats, due to factors including faster disease progression, and challenges in nutritional care (8).

Acute kidney injury (AKI) may result from renal insult caused by nephrotoxins such as heavy metals, drugs, plants, as well as infection and ischaemia. This sudden decrease in kidney function can lead to progression of CKD, although actual prevalence or incidence of progression from AKI is unknown in companion animal species (9).

In veterinary medicine, CKD diagnosis is often challenging due to vague clinical signs. Statistically significant clinical signs in dogs include halitosis, anaemia, weight loss, cachexia, polyuria/polydipsia, urinary incontinence and vomiting (4). In cats, clinical signs are similar and include vomiting, polyuria/polydipsia, decreased appetite, reduced energy levels and halitosis (6). It is well recognised that during stage 1 CKD, overt clinical signs are typically absent, and only subtle changes may be detected through laboratory analyses (10).

Biomarkers are widely used for disease diagnosis in veterinary medicine and can broadly be classified into diagnostic, prognostic, and predictive categories (11). Diagnostic biomarkers are of particular importance in veterinary medicine owing to the lack of pathognomonic and vague clinical signs especially in early stages of the disease. The combined use of multiple biomarkers rather than reliance on a single marker is increasingly employed to enhance diagnostic accuracy. Prognostic biomarkers can facilitate early disease detection and enable disease staging and treatment monitoring, whereas predictive biomarkers utilise advanced analytical approaches to assess disease progression or therapeutic response (11).

However, some traditional biomarkers have limited ability to detect disease progression and predict outcomes (12). Consequently, novel biomarkers continue to be developed to improve diagnostic and prognostic accuracy (12).

In human CKD, circulating biomarkers currently used in clinical practice have limitations in sensitivity and specificity for early disease detection and prognostication. Encouragingly some emerging biomarkers exhibit greater stability and more disease specific expression patterns (13). Consequently, continued efforts to refine existing biomarkers and identify novel biomarkers and biomarker panels that more accurately reflect CKD diagnosis and progression remain essential in veterinary medicine (14).

This review summarises and discusses current approaches to the diagnosis, prognosis and prediction of CKD progression, as well as their potential use in companion animals.

## 2 Diagnosis of CKD

The diagnosis of CKD in companion animals involves a combination of clinical history, physical examination and clinical pathology (blood and urine analyses). Additional examinations such as blood pressure, urine bacterial cultures and advanced imaging may also be required (8, 15). Blood biomarkers can assist in the assessment of CKD progression; for example, symmetric dimethylarginine (SDMA) demonstrates greater sensitivity than serum creatinine as an indirect marker of glomerular filtration rate (GFR). However, evidence of renal abnormalities may still be present in the absence of detectable changes in SDMA concentrations, highlighting the need for more precise and sensitive biomarkers in veterinary medicine (1, 2).

The International Renal Interest Society, IRIS, recommends the diagnosis and staging of CKD using markers such as serum creatinine, SDMA, GFR and uACR (Table 1) (10). A combination of these indicators determines the overall stage of CKD. Once this has been established, treatment options can be identified. However, the use of these markers in companion animals for example, in the determination of eGFR largely varies widely depending on the animal’s breed, size and body weight. In addition, because of the cost and technical difficulty associated with measuring eGFR is not widely adopted in routine veterinary practice (16, 17). Reductions in muscle mass can lower serum creatinine concentrations, potentially leading to inaccurate diagnostic interpretations (18). Furthermore, both urea and creatinine lack sufficient sensitivity for detecting kidney disease at its early stages, which may delay diagnosis and the initiation of appropriate therapeutic interventions, thereby allowing further disease progression (18). Overall, other limitations associated with the determination of GFR include the potential for errors arising from the use of small plasma samples, for example in anaemic animals. In addition, standardisation is required to account for differences between pet breeds; however, the optimal method of standardisation remains unknown. Creatinine, on the other hand, is influenced by metabolic factors, and its underestimation may therefore lead to inaccurate results. Furthermore, variation related to the timing of sample collection also requires careful consideration (19).

**Table 1:** IRIS reference ranges for feline and canine CKD markers used to diagnose and stratify.

CKD stages	Category	Creatinine^a^^,^^b^		SDMA^a^ μg/dL	GFR mL/min/kg	uACR mg/g	Species
		μmol/L	mg/dL				
	Healthy	n/a	n/a	n/a	2.66–5.67^c^	2.1^f^	Canine
		n/a	n/a	n/a	2.18–6.01^d^	5.5 ± 3.9^g^	Feline
1	Normal	<125	<1.4	<18	n/a	54.2 (early stages)^f^	Canine
		<140	<1.6	<18	1.21–4.60^d^	24.2 ± 18.0^g^	Feline
2	Mid azotemia	125–250	1.4–2.8	18–35	2.4^e^	n/a	Canine
		140–250	1.6–2.8	18–25	0.68–2.72^d^	21.3 ± 39.5^g^	Feline
3	Moderate azotemia	180–440	2.1–5.0	36–54	n/a	n/a	Canine
		250–440	2.9–5.0	26–38	0.66–2.13^d^	38.0 ± 55.4^g^	Feline
4	Severe azotemia	>440	>5.0	>54	1.5^e^	n/a	Canine
		>440	>5.0	>38	0.28–1.64^d^	80.4 ± 63.4^g^	Feline

a International Renal Interest Society (10).

b Bartges (8).

c Kampa et al. (109).

d Iwama et al. (110).

e Cortadellas et al. (111).

f Paukner et al. (41).

g Cha et al. (112).

n/a, non available.

In an effort to facilitate the early detection of CKD by owners, a study reported that polydipsia and polyuria were among clinical signs recognised by pet owners and observed several years prior to diagnosis. In dogs, owners were able to detect other signs, such as weight loss, approximately 4 months before a diagnosis of CKD (20). A limitation of this study is that pets included were geriatric animals, and a difference in body weight existed between the control group and the CKD cases. Furthermore, additional factors, such as diet and breed, may have acted as confounding and should therefore be considered when interpreting the clinical signs and study findings.

## 3 Clinical management and decision making in CKD

The management of CKD in veterinary medicine generally focuses on supportive therapy rather than curative treatment (21). Strategies are mainly aimed at treating complications and comorbidities such as hypercalcaemia, cardiac disease, periodontal disease, cystitis and urolithiasis (1). Treatment is generally based on nutrition, hydration and management of complications and sequelae (1, 2), with the objective of maintaining phosphate concentration levels and urine protein creatinine ratio. Supplements containing, for example, sodium bicarbonate, calcium carbonate, calcium lactate-gluconate and chitosan, administered once daily, have been shown to slow the progression of CKD (22). In addition, some diets are based on reduced protein content with high caloric density and are enriched with vitamins, antioxidants and other supplements (21). Hypertension is common in CKD so other specific treatments focus on controlling systolic blood pressure, such as angiotensin-converting enzyme (ACE) inhibitors, angiotensin II receptor blockers, and calcium channel blockers (23) hyperphosphatemia is treated with phosphate binders such as aluminium hydroxide or calcium carbonate, while proteinuria with angiotensin receptor blockers and antithrombotic agents. To reduce phosphate intake and maintain appropriate plasma phosphate concentrations, phosphate-binding therapy is recommended. Lanthanum carbonate has demonstrated efficacy and was recently approved by the European Commission (24). Ferric citrate has also been recommended for the management of CKD in dogs (25). Ferric citrate has been associated with improvements in kidney function, reductions in albuminuria, and decreased inflammation (24, 26). Anti-nausea medications and appetite stimulants are also commonly used in the management of CKD (23). Both cats and dogs are recommended to be re-evaluated every 3 to 6 months, even if they appear clinically stable (10). Adequate hydration, dietary management and the control of bone mineral disorders are particularly important in cats. Renal diets have been shown to significantly increase longevity, more than doubling survival time in cats (27).

## 4 Biomarkers

Biomarkers are objectively measured, quantifiable indicators of biological processes related to either health, disease, or response to interventions. Ideally, these can be measured by non-invasive or minimally invasive methods and in CKD are used for detecting organ damage and dysfunction (28). In human and veterinary CKD biomarker diagnostic assessment is conducted via assessment of serum and urine.

Urinary markers can reflect renal damage while others may indicate glomerular and tubular dysfunction. In the dog, immunoglobulin G (IgG), C reactive protein (CRP) and podocin, are recognised markers of glomerular damage (28). These markers can be detected in urine and may also reflect other disease processes, such as AKI, parvovirus, leishmaniasis and degenerative mitral valve disease (28). Podocin in particular, appears to be an indicator of podocyte loss and active glomerular injury (28). Measurements of podocin in dogs have shown that those with cardiorenal syndrome, for example have a podocin creatinine ratio of 9.7 ± 4.8 ×10^−10^ in control dogs, compared with 33.7 ± 18.0 ×10^−10^ in azotemic dogs (29).

The evaluation of urine enzyme activity can be used to assess tubular damage and dysfunction. For example, an increase in the levels of Gamma-Glutamyl Transferase (GGT), Alkaline phosphatase (ALP), and n-Acetyl Beta D- Glucosaminidase (NAG) are associated to tubular damage. However further studies are required to confirm their value in clinical decision-making processes (28). In dogs, urinary GGT has been used to evaluate active tubular injury; however, its activity is affected by urine pH, and sample contamination with blood or bacteria (30). In cats, the evaluation of biomarkers for tubular damage remains limited and requires further studies involving statistically significant and controlled studies (31). Some of these enzymes can also be measured in blood; however, their interpretation may be limited because their concentrations can be influenced by diseases other than renal pathology. For example, GGT and ALP are routinely measured in serum as indicators of liver function (30, 32).

Some markers can be detected in serum while others are found in urine, and several have been implicated in the alteration of renal disease. These include Neutrophil Gelatinase Associated Lipocalin (NGAL), Cystatin B and C (CysB, CysC), clusterin, Kidney Injury Molecule 1(KIM-1), Retinol Binding Protein (RBP), Tamm-Horsfall protein or uromodulin (THP), Liver-type Fatty Acid Binding Protein (L-FABP), and Heat Shock Protein-72 (HSP-72) have been studied and implicated in the alteration of renal disease (28). Among these biomarkers, cystatin C has been proposed as a complementary biomarker for the detection of CKD in dogs and cats. However, its use is limited by challenges associated with its detection. In contrast, it may be more relevant for the diagnosis of AKI, as demonstrated by this meta-analysis, which showed high diagnostic accuracy in differentiating cats with AKI from healthy controls (33). Serum cystatin C in cats has a median reference interval between 0.58–1.95 mg/L (34). Cystatin B is now also a commercially available urinary test in dogs and cats and may represent a more reliable biomarker than traditional indices of kidney injury, however it does not confer any information of function (35).

The SMDA is another marker that aids in the detection of renal dysfunction, including CKD, in companion animals and has been incorporated into the IRIS guidelines (10). For example, in cats SDMA has been reported to demonstrate a sensitivity of 100% and a specificity of 91% (36). The usefulness of creatinine as a comparator biomarker is limited by its dependence on muscle mass, which may result in misleading values in heavily muscled animals. In contrast, SDMA concentrations remain relatively stable with increasing lean body mass (37). Body mass and composition vary significantly between breeds in both cats and dogs (38). For instance, SDMA concentrations are higher in greyhounds than in other canine breeds, likely due to their greater muscle mass, while Birman cats have been reported to possess an upper reference limit for SDMA higher than that typically cited for the broader species. These breed-associated differences affect SDMA interpretation as a universal renal biomarker. Additionally, age-associated changes in renal physiology and protein turnover demand age-stratified reference intervals to enable precise analytical interpretation and minimise misclassification in clinical settings (39).

Although SDMA analysis requires specialised equipment, many veterinary practices perform the test in-house using dedicated analysers, while others submit samples to external laboratories (10, 40). Albuminuria concentrations have been demonstrated to agree with those of SDMA levels, suggesting that the urinary albumin to creatinine ratio is as useful biomarker of CKD in canine patients (41).

In any new assay, be it derived and developed from information taken from human diagnostics, the following criteria need to be considered (Figure 1).

**Figure 1:**
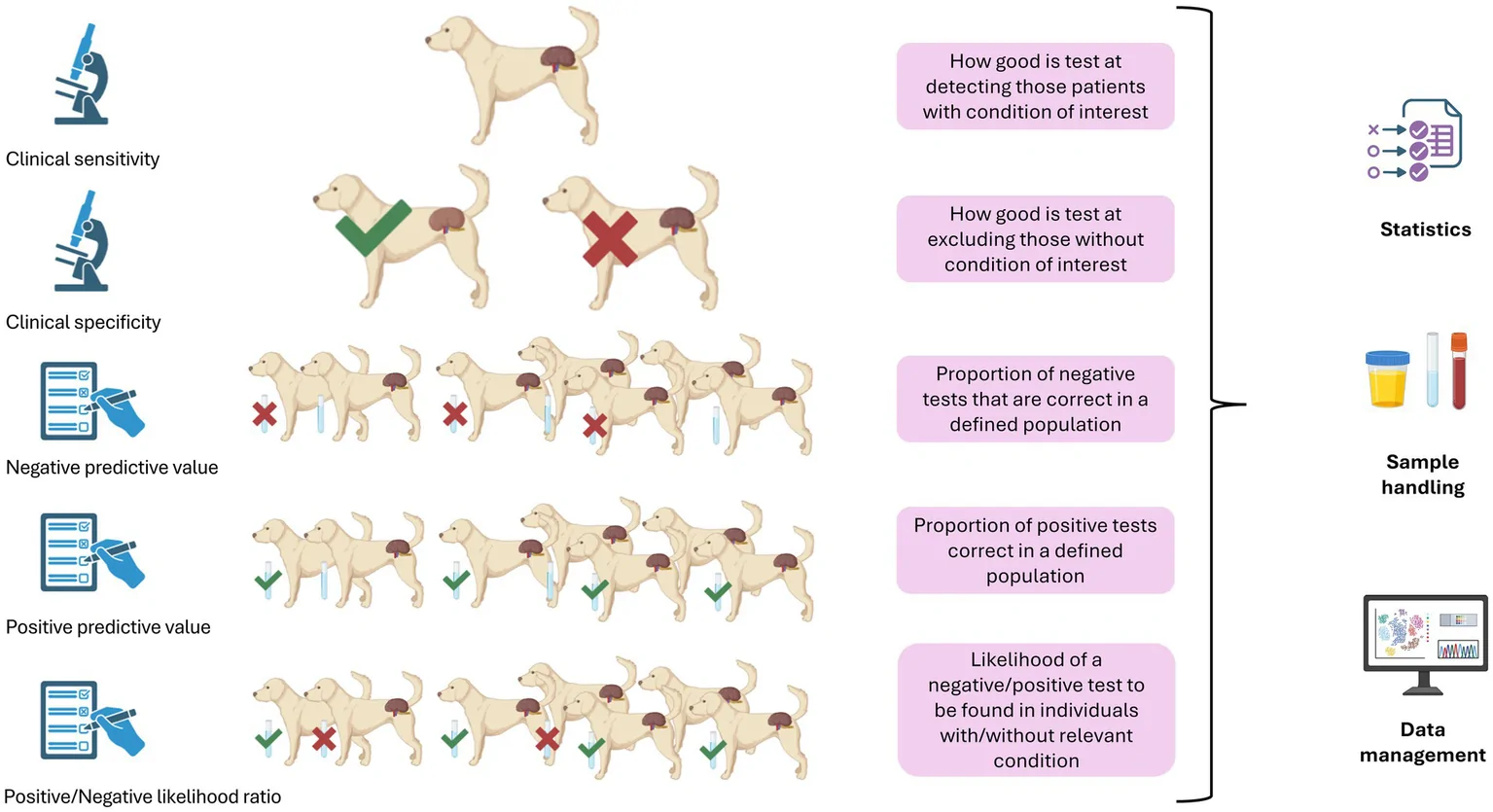
Key facets for the application of biomarkers in veterinary medicine. Appropriately designed studies should address or include: matched controls; study size for statistical powering; sample handling and storage consistency; data management; limit of detection; changes in analyte indicative of disease trajectory.

### 4.1 Genomics

The application of genomics in veterinary medicine has highlighted the importance of investigating the genetic basis of disease, improving the understanding of disease susceptibility and supporting more accurate diagnosis. In veterinary pharmacology, genomics has gained increasing interest because of its applications in drug development, pathogen detection, disease diagnosis, and the monitoring of drug residues and food safety (42). These technologies have also been increasingly applied in large animal species (14).

Polymorphisms associated with gene expression have been investigated in companion animals to identify disease risk and predisposition. For example, in German Shepherd dogs, specific single nucleotide polymorphism alleles have been shown to be associated with inflammatory bowel disease (43). Cystinuria in Newfoundland dogs has been shown to be homologous to human cystinuria, with mutations identified in the *SLC3A1* gene. In contrast, cystinuria in other dog breeds is genetically heterogeneous and may involve different genetic variants and modes of inheritance (44).

Genome wide association studies (GWAS) have revealed numerous loci for kidney function. Genotyping of Boxer dog populations from different countries identified 21 genomic loci associated with CKD. The strongest association signals were detected in regions containing the candidate genes *RELN* and *MAGI2*, both of which have previously been implicated in kidney development or disease. Other associated regions contained genes including *FGFR2* and *GALNT18*, which are involved in tissue development and cellular regulation (45).

Studies of genomic markers in conjunction with protein and metabolite biomarkers are not only valuable for the identification of diseases and for informing prognosis, diagnosis, and treatment but also for advancing research into other diseases (46). These analyses and developments in this field may also contribute to future research and clinical management in companion animals.

### 4.2 MiRNAs

One of the most promising novel biomarker types being extensively researched across species are non-coding RNAs such as microRNAs (miRNAs). MiRNAs are single stranded and are highly conserved across species making them valuable for comparative medicine analyses (47). They are reported to play a crucial regulatory function in kidney disease and are also implicated in key pathological processes such as renal fibrosis (48). MicroRNAs can be found in tissues and in biofluids, or enclosed within extracellular vesicles (EVs), which are released by cells and participate in intercellular communication (49). Additionally, miRNAs have been identified as potential diagnostic (Table 2) and prognostic indicators in CKD (47, 50). RNA sequencing analyses of exosomes or extracellular vesicles (EVs) isolated from urine samples have identified a range of non-coding RNAs, including miRNAs, long intergenic non-coding RNAs (lincRNAs), small nucleolar RNAs (snoRNAs), transfer RNAs (tRNAs), and small nuclear RNAs (snRNAs). The highest component of non-coding RNAs are miRNAs with ~78% of the reads in the urine of CKD from human urine (51). However, the exosomal abundance of non-coding RNAs varies across CKD stages, for example, levels of non-coding RNAs have been reported to be higher at stage 3 in CKD compared with healthy individuals (52, 53).

**Table 2:** MiRNAs biomarkers for the detection of renal condition in human and companion animals.

Localization	miRNA	Clinical significance	Expression	Species	Disease	Reference
Serum	*miR10b*	Prognostic	Decreased	*Canis lupus*	Kidney damage	(113)
	*miR-21* *miR-21a*	Prognostic	Decreased	*Canis lupus and Homo sapiens*	Kidney damage CKD (stage 1–4)	(113, 114)
	*miR-29a*	Prognostic	Decreased	*Homo sapiens*	CKD (stage 1–4)	(114)
	*miR-93*	Prognostic	Decreased	*Homo sapiens*	CKD (stage 1–4)	(114)
	*miR-107*	Diagnostic and prognostic	n/a	*Canis lupus*	Glomerular disease	(115)
	*miR-126*	Diagnostic Prognostic	Decreased	*Homo sapiens*	CKD (stage 3–5) CKD (stage 1–5)	(116, 117)
	*miR-129*	Diagnostic and prognostic	n/a	*Canis lupus*	Glomerular disease	(115)
	*miR-146a*	Prognostic	Decreased	*Canis lupus*	Kidney damage	(113)
	*miR-146*	Prognostic	Increased	*Felis catus*	Induced CKD	(55)
	*miR-155*	Prognostic	Increased	*Homo sapiens*	CKD (stage 1–4)	(114)
	*miR-186*	Diagnostic and prognostic	n/a	*Canine*	Glomerular disease	(115)
	*miR-190*	Diagnostic and prognostic	Decreased	*Homo sapiens*	CKD	(53)
	*miR-197*	Diagnostic	Decreased	*Homo sapiens*	CKD (stage 3–5)	(116)
	*miR-199*	Prognostic	Increased	*Felis catus*	Induced CKD	(55)
	*miR-200a*	Prognostic	Increased	*Homo sapiens*	CKD (stage 1–4)	(114)
	*miR-214*	Prognostic	Increased	*Homo sapiens*	CKD (stage 1–4)	(114)
	*miR-223*	Diagnostic Prognostic	Decreased	*Homo sapiens*	CKD (stage 3–5) CKD (stage 1–4)	(114, 116)
	*miR-365* *miR-371*	Diagnostic and prognostic	n/a	*Canis lupus*	Glomerular disease	(115)
	*miR-382*	Prognostic	Decreased	*Felis catus*	Induced CKD	(55)
	*miR-486*	Prognostic	Decreased	*Homo sapiens*	CKD (stage 1–4)	(114)
	*miR-7* *miR-9*	Diagnostic and prognostic	n/a	*Canis lupus*	Glomerular disease	(115)
Urine	*miR10a*	Prognostic	Low in urine exosomes	*Canis lupus*	Kidney damage	(113)
	*miR-21a* *miR-21*	Prognostic Diagnostic and prognostic	High in urine exosomes	*Canis lupus and Felis catus*	Kidney damage	(62, 113, 115, 118)
	*miR-22* *miR-22*	Diagnostic and prognostic	Decreased	*Canis Lupus* *Felis catus*	Glomerular disease In cats at early stages of kidney disease	(115, 118)
	*miR-26a*	Prognostic	Low in urine exosomes	*Canis lupus* *Felis catus*	Kidney damage; CKD In cats at early stages of kidney disease	(113, 118)
	*miR-29*	Prognostic		*Homo sapiens*	CKD (stage 1–4)	(114)
	*miR-30e*	Diagnostic	Downregulated	*Felis catus*	CKD	(62)
	*miR-33*	Diagnostic	Upregulated	*Homo sapiens*	CKD	(61)
	*miR-93*	Prognostic	Decreased	*Homo sapiens*	CKD (stage 1–4)	(114)
	*miR-126*	Diagnostic and prognostic	Increased	*Canis lupus*	Amyloidosis, glomerulosclerosis, immune complex-mediated glomerulonephritis	(115)
	*miR-146a*	Prognostic	High in urine exosomes	*Canis lupus*	Kidney damage	(113)
	*miR-155*	Prognostic	Decreased	*Homo sapiens*	CKD (stage 1–4)	(114)
	*miR-182*	Diagnostic and prognostic	n/a	*Canis lupus*	Amyloidosis, glomerulosclerosis, immune complex-mediated glomerulonephritis	(115)
	*miR-190*	Diagnostic	Upregulated	*Homo sapiens*	CKD	(61)
	*miR-191*	Prognostic	Low in urine exosomes	*Canis lupus*	Kidney damage	(113)
	*miR-194*	Diagnostic	Downregulated	*Felis catus*	CKD	(62)
	*miR-196* *miR-200*	Prognostic	Increased Decreased	*Homo sapiens*	CKD (stage 1–4)	(114)
	*miR-203*	Diagnostic and prognostic	n/a	*Canis lupus*	Glomerular disease	(115)
	*miR-214*	Prognostic	n/a	*Homo sapiens*	CKD (stage 1–4)	(114)
	*miR-423a*	Diagnostic and prognostic	n/a	*Canis lupus*	Glomerular disease	(115)
	*miR-507*	Diagnostic	Upregulated	*Homo sapiens*	CKD	(61)
	*miR-542*	Diagnostic	Upregulated	*Homo sapiens*	CKD	(61)
	*miR-10a* *miR-10b*	Prognostic	n/a	*Canis lupus*	Kidney damage	(113)
Kidney tissue	*miR-21* *miR-21a* *miR-21*	Diagnostic Prognostic Prognostic	High expression Upregulated	*Homo sapiens* *Canis lupus Canis lupus* *Felis catus*	CKD Kidney damage Dogs with XLHN hereditary nephropathy Induced CKD	(54, 55, 113, 119)
	*miR-26b*	Prognostic	n/a	*Canis lupus*	Dogs with XLHN hereditary nephropathy	(54)
	*miR-30d*	Prognostic	Downregulated	*Homo sapiens*	Progressive CKD	(120)
	*miR-140*	Prognostic	Downregulated	*Homo sapiens*	Progressive CKD	(120)
	*miR-142* *miR-146* *miR-147*	Prognostic	Upregulated Upregulated Upregulated	*Canis lupus*	Dogs with XLHN hereditary nephropathy	(54)
	*miR-186*	Prognostic	n/a	*Canis lupus*	Dogs with XLHN hereditary nephropathy	(54)
	*miR-190* and *194*	Prognostic	Downregulated	*Homo sapiens*	Progressive CKD	(120)
	*miR-199*	Prognostic	Increased	*Felis catus*	Induced CKD	(55)
	*miR-205* *miR-206*	Diagnostic Prognostic	Downregulated	*Homo sampiens*	CKD and progressive CKD	(119, 120)
	*miR-235* *miR-382*	Prognostic	Increased	*Felis catus*	Induced CKD	(55)
	*miR-802*	Prognostic	Upregulated	*Canis lupus*	Dogs with XLHN hereditary nephropathy	(54)

n/a, non available.

Among the various miRNAs investigated in the context of CKD, several are markedly expressed and exhibit strong potential as diagnostic or prognostic biomarkers. One miRNA of significant interest in CKD diagnosis and treatment is Mir-21. It is expressed both within kidney and in circulating biofluids (see Table 2). MiR-21 was consistently reported to be significantly upregulated in the kidneys of dogs with chronic kidney disease at different stages of disease, as identified using three sequencing analysis platforms (miRDeep2, CPSS 2.0, and OmicSoft Studio) (54). Another study demonstrated the relevance of miR-21 in feline CKD, reporting increased expression of miR-21 in both renal tissue and serum samples from cats with experimentally induced disease (55). Histopathological evaluation in the previously mentioned studies revealed renal fibrotic changes in both dogs and cats, while inflammation and tubular atrophy in the feline study were assessed using a semi-quantitative scoring system ranging from 1 to 3. The involvement of miR-21 in fibrotic kidney disease is further supported by extensive human research, which has demonstrated a key role for miR-21 in renal fibrogenesis. Consequently, silencing or knockdown of miR-21-5p was proposed as a potential therapeutic approach to kidney fibrosis through modulation of TGF-β1 signalling (56, 57).

However, a recent clinical trial in humans evaluating the anti-miR-21 oligonucleotide lademirsen in adults with Alport syndrome reported no significant differences in eGFR between treatment and placebo groups, highlighting the need for further clinical studies to establish its therapeutic efficacy (58). Dogs can develop X-linked hereditary nephropathy (XLHN), which is considered the canine counterpart of human Alport syndrome (59). Increased miR-21 expression has been observed in affected dogs at various stages of XLHN disease, indicating its potential involvement in the pathogenesis and progression of CKD (59). MiR-21 cannot be regarded as a standalone biomarker because it is expressed in a range of tissues and other diseases and its clinical utility has not progressed beyond early-stage clinical trials, likely in part due to a lack of clinical specificity (60). MiR-29 is another miRNA that expressed in XLHN-related renal injury, it appears to be upregulated during the early stages of proteinuria, whereas downregulation occurs with the onset of azotaemia (54). Expression of the miR-29 family also decreases during CKD progression. Beneficial effects such as attenuation of renal fibrosis and reduced blood urea have been found when injecting exosomes containing the miR-29 family in mice with unilateral ureteral obstruction (56, 57).

In addition to the miRNAs discussed above, miR-190 appears to be a promising prognostic biomarker for CKD in humans, with reduced circulating levels being associated with impaired kidney function (61). Another recent study demonstrated that circulating miR-190 may aid in predicting CKD progression, as its expression correlates with that of eGFR in humans, with this relationship remaining independent of diabetes status (53). To validate the association between miR-190 and kidney function, that same study correlated its expression with biopsy findings and tubular cell immunostaining, confirming a strong renal contribution to circulating levels (53). However, to the best of our knowledge, its expression in urine and serum has not been investigated as extensively in dogs and cats as it has in humans. Table 2 provides an overview of other miRNAs studied in renal diseases.

Compared with human medicine, relatively few transcriptomic studies investigating microRNAs in canine and feline urinary tract and kidney diseases have been reported. Godia et al. (62) identified 25 differentially abundant miRNAs associated with CKD compared to healthy cats; of these 51 of these genes were shared between pyelonephritis and other urological conditions. In cats with experimentally induced CKD, Grimes et al. reported increased expression of four miRNAs, in both ischaemic renal tissue and serum, whereas one miRNA exhibited opposite expression patterns, being upregulated in ischaemic kidneys but downregulated in serum (55). In dogs affected by XLHN that developed CKD, up to 23 miRNAs were differentially expressed compared with controls, several of which were consistently identified across different analytical approaches (54).

The use of microRNAs as biomarkers are not without limitations, such as potential temporal variations in circulating microRNA levels, which means that the timing of sample collection must be standardised when detecting disease. Appropriate sample preservation is also essential to prevent microRNA degradation (63). Further research in this area in companion animals is required, as the available data is currently limited.

### 4.3 Transcriptomics and biomarker discovery in CKD

Investigating gene expression through RNA sequencing and microarrays helps identify a large number of putatively pathogenic mechanism-related gene products and elucidate the pathways involved in the disease. Transcriptomics aids our understanding of the molecular mechanisms of CKD but also introduces novel ideas for diagnostic/treatment approaches (64). Single-cell RNA sequencing is one approach which measures gene expression at the individual cell level to identify variation in cell types and populations. Cell-specific transcriptional changes have become pivotal for mapping cellular interactions, identifying the cell types involved in disease progression, and understanding how each defined cell type contributes to pathophysiology (65).

A microarray analysis of the renal cortex in dogs revealed that dogs with X-linked agammaglobulinemia (XLA) kidney showed increased expression of genes involved in cell migration and inhibition of protein synthesis. Dogs affected by this disease also showed higher expression of genes associated with collagen production, which may contribute to extracellular matrix remodelling within the kidney tissue (66). RNA sequencing of serial kidney biopsies from dogs with XLHN identified differential expression of genes and pathways associated with disease progression, including integrin signalling, T-cell activation, and chemokine and cytokine-mediated immune responses (67). Single-cell RNA sequencing of kidney tissue from dogs with advanced to end-stage XLHN identified cell type-specific gene expression changes, including enrichment of the integrin signalling pathway and inflammatory pathways mediated by chemokine and cytokine signalling in proximal tubule cell subpopulations (68).

Spatial transcriptomics offers valuable insights into cellular organisation and the localised differences in disease-associated gene expression (69). It can therefore help reveal molecular changes that occur during disease progression and may be particularly useful for identifying early-stage disease. When combined with other techniques, such as proteomics and epigenetics, it contributes to a more comprehensive and spatially resolved understanding of disease processes (70).

These studies have provided valuable insights into the molecular changes occurring within renal tissue. However, the identification of circulating biomarkers could facilitate the assessment of disease progression using minimally invasive biological samples obtained from large populations of companion animals, thereby improving our understanding of CKD progression without affecting animal welfare.

### 4.4 Proteomics and biomarker discovery in CKD

Similar to transcriptomic analysis, proteomics generates rich datasets that enable in-depth analysis of potential biomarker signatures. These can be integrated with transcriptomic and metabolomic data to provide a comprehensive overview of CKD pathophysiology (Table 3). Moreover, proteomics, more so than other omics such as genomics and transcriptomics, provides a real-time molecular “snapshot” of a patient which is closer to the phenotype as it measures the functional molecules directly responsible for biological processes. Proteomic studies in CKD have identified potential protein biomarkers at different stages of the disease, based on analyses of blood and urine samples. In human studies, common biomarkers used for the detection of CKD such as urea and glucose behave differently at different CKD stages (71). These variations correlate with significant differences observed in the proteome, with several proteins showing differential expression in CKD IRIS stages 1–3 compared with healthy individuals. In cats with CKD a proteomic analysis revealed that proteins involved in the glutamine metabolism were altered. Proteins associated with fibrosis and tissue remodelling were increased, and changes in proteins involved in oxygen homeostasis were observed, with these alterations being more pronounced in cats with CKD stages 3–4 than in those with stages 1–2 (72).

**Table 3:** Protein markers identified from proteomic analysis of the kidney.

Sample type	Proteomic markers	Clinical significance	Expression	Species	Disease	Reference
Kidney	Retinoic acid receptor responder protein 2, C-C motif chemokine 21, Coiled-coil domain-containing protein 80	Diagnostic and prognostic	Increased	*Homo sapiens*	Diabetic kidney disease	(121)
Urine	Vitamin D–binding protein, serum albumin, Zinc-alpha-2-glycoprotein, Immunoglobulin kappa constant region, fetuin-A, Alpha-1B-glycoprotein, transferrin, and IgA2 heavy chain	Diagnostic	Increased (in kidney damage)	*Homo sapiens*	CKD	(122)
	Osteoponin, CD59, uromodulin, Kininogen-1 Ribonuclease 1, CD44, Alpha-1-microglobulin/bikunin precursor, Zinc-alpha-2-glycoprotein and Prostaglandin-D2 synthase	Diagnostic	Decreased (in less kidney damage)	*Homo sapiens*	CKD	(122)
	Cluster of differentiation 44. Vitamin D–binding protein.	Diagnostic	Increased (eGFR-in kidney damage). Decrease (associated to eGFR in kidney preserved function).	*Homo sapiens*	CKD	(122)
	Serum amyloid A1	Diagnostic	Increased in AKI	*Homo sapiens*	AKI	(123)
	Complement component 5, Apolipoprotein C-I, and Regenerating islet-derived protein 3 alpha	Diagnostic	Increased in AKI and CKD	*Homo sapiens*	AKI	(123)
	Retinol binding protein 4. Hemopexin, beta-2-microglobulin, transthyretin and factor D.	Diagnostic	Increased associated with glomerular disease. Increased.	*Homo sapiens*	ESRD (End Stage Renal Disease)	(124)
	Neutrophil gelatinase-associated lipocalin	Diagnostic and predictive	Increased concentration	*Homo sapiens*	AKI	(125)
	Collagen-alpha-1, and uromodulin	Diagnostic	n/a	*Canis lupus*	CKD	(126)
	Uromodulin	Diagnostic	Decreased	*Canis lupus*	CKD	(127)
	Kidney Injury Molecule-1, Neutrophil Gelatinase-Associated Lipocalin, Clusterin, osteoactivin, collagen type 1 alpha 1 chain, calbindin, vascular endothelial growth factor, tissue inhibitor of metalloproteinases-1, matrix and metalloproteinase 9, tissue inhibitor of metalloproteinases-1	Prognostic and predictive	Biomarker expression panel for the prediction of kidney failure/all-cause mortality	*Homo sapiens*	Kidney Failure or all-cause mortality	(81)
	Vascular endothelial growth factor, Tissue Inhibitor of Metalloproteinases-1, collagen type 1 alpha 1 chain	Prognostic and predictive	Upregulated in males	*Homo sapiens*	CKD	(128)
	Osteoactivin, metalloproteinase 9, Kidney Injury Molecule-1 and fibroblast growth factor 23	Prognostic and predictive	Upregulated in females	*Homo sapiens*	CKD	(128)
Plasma	Whey Acidic Protein four-disulfide core domain protein, twisted gastrulation follistatin-related protein 3, transmembrane emp24 domain containing protein 2	Prognosis	Increased in kidney risk inflammatory signature. Decreased in kidney risk inflammatory signature.	*Homo sapiens*	CKD	(129)
	Myc proto-oncogene protein, zinc finger protein 329, interleukin-5, selenoprotein H, corticotropin	Prognostic	Decreased in kidney risk inflammatory signature	*Homo sapiens*	CKD	(129)
	Metalloproteinase 7,	Diagnostic and prognostic	Concentration increased	*Homo sapiens*	Diabetic kidney disease	(121)
	Chaperonin Containing TCP1 Subunit 4, Coiled-Coil Domain Containing 25, 3-Hydroxy-3-Methylglutaryl-CoA Synthase 1, Cytosolic, Immunoglobulin Heavy Constant Mu, Protein Disulfide-Isomerase A4, Plasminogen Activator Inhibitor 1 RNA Binding Protein, Rho GDP-Dissociation Inhibitor Beta, Mediator Complex Subunit 30, Tenascin XB, Heterogeneous Nuclear Ribonucleoprotein H1	Prognostic	Upregulated	*Homo sapiens*	CKD	(130)
	Afamin, Transthyretin, Complement Component 6, Retinoic Acid Receptor Responder 2 / Chemerin, FK506 Binding Protein 4, Proteasome Subunit Beta Type-5, Protein Disulfide-Isomerase A4, Vimentin, Complement C1q A Chain, C4b-Binding Protein Beta Chain, Myosin Light Chain Kinase, Syntaxin-Binding Protein 1 / Munc18-1, Kallistatin	Prognostic	Downregula-ted	*Homo sapiens*	CKD	(130)
	Neutrophil Gelatinase-Associated Lipocalin, cluster of differentiation 40, soluble Tumour Necrosis Factor Receptor 1, Monocyte Chemoattractant Protein-1, cardiac troponin-T, Kidney Injury Molecule-1, growth differentiation factor 15, pro-B-type natriuretic peptide	Prognostic and predictive	Upregulated in males	*Homo sapiens*	CKD	(128)
	Osteoactivin, matrix and metalloproteinase 9	Prognostic and predictive	Upregulated in females	*Homo sapiens*	CKD	(128)
Serum	Neutrophil Gelatinase-Associated Lipocalin	Diagnostic and predictive	Increase concentration	*Homo sapiens*	AKI	(125)
	Serpin family F member 1, Cathepsin B, Tre-2, Budding Uninhibited by Benzimidazoles 2, Cell Division Cycle 16, and TBC1 Domain Family Member 4	Diagnostic and predictive	Increased	*Felis catus*	CKD	(131)
	Kidney Injury Molecule-1, Neutrophil Gelatinase-Associated Lipocalin clusterin, soluble tumour necrosis factor receptor 1, cluster of differentiation 40, urokinase plasminogen activator receptor, monocyte chemoattractant protein-1, high sensitivity cardiac troponin-T, growth differentiation factor 15, fibroblast growth factor 23	Prognostic and predictive	Biomarker expression panel for the prediction of kidney failure/all-cause mortality in plasma/serum	*Homo sapiens*	Kidney Failure or all-cause mortality	(81)

n/a, non available.

The limitations of using proteomics for biomarker detection include the need, with some methods, for strict temperature control during sample handling to preserve protein stability (73). In addition, metabolic changes can occur in blood samples over time. Clinical information from the patient is also required to enable correlation of proteomic findings with the presence of other proteins and underlying conditions (73). Further research in veterinary medicine, particularly in CKD, is needed, as most companion animal proteomic studies aimed at biomarker detection have primarily focused on infectious diseases and have mainly used serum and saliva samples (74).

A study in humans showed that the levels of biomarkers such as kidney injury molecule (KIM-1), interleukin 18 (IL-8) and monocyte chemoattractant protein (MCP-1) are strongly correlated with urine creatinine and urine osmolarity. This correlation remains important for the normalisation or indexing of biomarkers or development of predictive algorithms with greater sensitivity and specificity. However, such strong correlation differences are only observed in some biomarkers, particularly in those originated from the proximal tubules (75).

As most of the available evidence on genomic, transcriptomic and proteomic biomarkers is derived from human studies, a summary of the key molecular pathways implicated in kidney disease progression is presented below (Figure 2).

**Figure 2:**
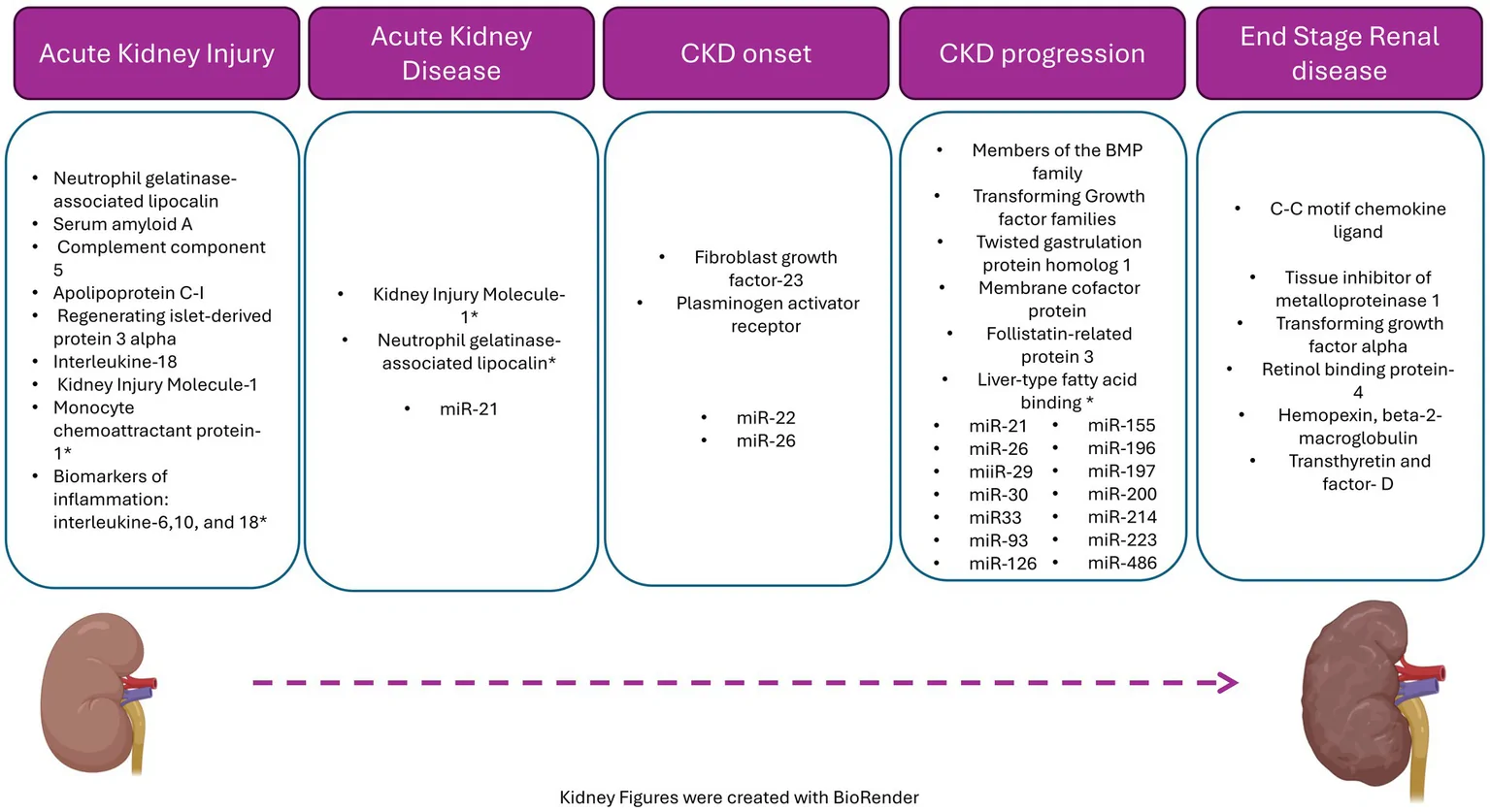
Summary of biomarkers identified biofluids in human and veterinary -omics studies as being associated with kidney disease. The boxes highlight the main biomarkers reported in the literature at different stages of CKD described in this manuscript. * Biomarkers described by Munshi et al. (108) have been only presented in this figure. Kidneys were created with BioRender.com.

There is no “one size fits all” approach to validating biomarkers as they can vary from questionnaires to imaging data to protein/RNA measurements. Different validation strategies are required for DNA, RNA and protein-based assays and must adhere to the broad tenet of Good Clinical Practice guidelines (63, 76) whatever the species involved. In CKD this can be challenging, as the disease typically progress slowly, meaning longitudinal sample collection and potentially long clinical trials. This inherently requires repeated sampling from large numbers of well-stratified patients (often hundreds of specimens), which presents a significant challenge in veterinary medicine and necessitates the development of biobanking resources and collaborative networks supported by high-level health informatics. We have recently established such an infrastructure. Assay validation for use in the clinic beyond clinical trials requires access to accredited pathology laboratories (ISO15189/FDA) in human medicine, and equivalent standards should be met for companion animals. Otherwise, one may fail to meet the criteria Figure 1.

### 4.5 Biomarkers panels

The identification of specific genes in human studies has led to the development of biomarker panels that provide a broader understanding of the pathways and interactions underlying this disease. The development of a panel of biomarkers is of interest as it may help predict the risk of CKD in individual patients, given that biomarker expression varies with age (77). However, the measurement of multiple entities must lead to easily interpreted results (i.e., via an algorithm giving a simple score) and also have an appropriate cost level. Notably, among the biomarkers routinely measured in clinical practice, the most used reflect the status of specific regions within the kidney and help to assess disease severity.

Integrating omics analyses allows for the potentially more incisive assessment of CKD stage and enables prediction of the extent of kidney function impairment. The integration of data from genes associated with CKD together with multiomic datasets from larger reported studies can provide an overview of the pathology that in turn informs biomarker research and development. Data collection from catalogues, published findings, and other studies, together with bioinformatic tools and artificial intelligence approaches, helps to integrate potential lists of genes and gene products as biomarkers identified in other diseases. This strategy can also be highly relevant for research into the detection and treatment of CKD (78–80). The identification of a panels of protein biomarkers, as studied in humans may contribute to the development of risk prediction models for canine and feline kidney failure and kidney-related all-cause mortality (81). In addition to this, the evaluation of CKD in dogs suggest them as a comparative model for humans (82).

## 5 Dogs as models of human renal failure

To effectively discover, test and validate novel biomarkers, often research has leveraged animal models for renal disease as these offer easier access to samples as well as some methodological and ethical advantages. The selection of animal models for studies covers factors such as animal size, cost, availability of housing, confounding co-infections (83). Mice have long been used as animal models for human disease due to their similar organs and bodily systems, meaning they experience many of the same diseases as humans (84). As a result, animal models have greatly enhanced our knowledge and understanding of many human genetic diseases, while also providing opportunities for the discovery of novel therapeutic targets and gene function (85). However, the inability of mice to present some human disease symptoms means that treatments may not always translate between species, e.g., human Huntington’s Disease patients display dyskinesia, yet mice do not. Rodents have also been used in research because they are easy to handle and relatively inexpensive. However, their value as translational models is also limited by their short lifespan, and they may be more suitable for studying the genetic origins of a disease rather than its long-term progression (86). Therefore, the use of dogs can alleviate some of these discrepancies as they are genetically closer to humans. Furthermore, reverse translational research in naturally occurring canine disease exemplifies the One Health approach, benefiting both human and veterinary medicine and advancing biomedical science (86). In the case of dogs and CKD the use of canine species within appropriate ethical and legal frameworks will benefit canine health as well as human health in the medium and long term.

Whilst the structural anatomy of dog and human kidneys is not identical, research suggests the extensive similarities support the use of dogs as an animal model for human kidney disease (87, 88). In addition, concepts originating in human medicine have been successfully translated into veterinary practice in the context of kidney disease (89). Dogs are also considered as one of a few non-human species that have similar urinary tract anatomy comparable to humans, and they exhibit similar urinary behaviours (90). Although a relationship between CKD and diabetes in dogs may be plausible, overextension of modelling with canines should be avoided (91). Conversely, the study of geriatric dogs provides valuable insights into the natural evolution of pathology, which may aid earlier detection of CKD in humans (92).

According to the Online Mendelian Inheritance in Animals (OMIA) database, 636 genetic diseases in dogs have potential as models for human traits (93). Molecular mechanisms involved in genetic disease have been revealed during canine studies, e.g., the genetic basis for progressive myoclonic epilepsy, PME in miniature wirehaired dachshunds (94). Genetic studies in dogs are often simpler than those conducted in complex populations as individual represent relatively closed populations with limited loci and disease heterogeneity (95). Additionally, dogs have long stretches of linkage disequilibrium, meaning that fewer genetic markers are required for genome wide investigations. Dogs also share many common environmental factors to humans including diet, carcinogenic load, and immunologic profile, making them good models for human cancer research (95).

As an example, dogs are a useful model for Alport syndrome and progressive CKD in humans (54). Studies have identified differentially expressed miRNAs that could regulate critical pathways in CKD progression in this instance, providing much needed insights for human research.

Dogs are an excellent model for ageing as they have a significantly shorter life span than humans but an anatomy that is far more similar to humans than do mice (96). In addition to this, dogs share similarities with humans with respect to age and risk in neoplastic, congenital and metabolic death causes (97).

## 6 Naturally occurring models are better than artificial models

The advantages of naturally occurring disease models over artificial models include a development of conditions, chronologic age in which conditions develop, and similar environmental factors as outlined below. Renal-derived cells can be cultured in monolayers or two-dimensional systems; however, these cells lose their native characteristics under *in vitro* conditions. Alternative human kidney models such as 3D organoids derived from stem cells represent an innovative substitute for animal models; nevertheless, they present several limitations including poor vascularisation, absence of immune cells, and lack of glomeruli tubular flow (98). In veterinary medicine, the successful isolation of organoids from female canine tissues has shown that these *in vitro* cultures retain key molecular and histological characteristics of their tissue of origin. Gene expression profiles were comparable to those of the corresponding native tissues, while histological analyses identified parietal cells and distal tubules in kidney organoids and basal and umbrella cells in urinary bladder organoids (99). These models offer promising opportunities for translating experimental approaches developed in human medicine to veterinary research and clinical practice (98). Although certain limitations should be considered. Renal organoids seem to mimic tissue characteristics, however, they appear to lose their oxygen balance after culture passages and, when compared to tissue specific markers, exhibit higher expression levels than those observed in renal tissue (100). In veterinary medicine, the standardisation of organoid methodologies is still in progress. Further standardisation is required to ensure reproducibility in research, facilitate comparisons between studies, and advance the field (86).

Among the advantages of natural and artificial models for the study of renal failure are:

### 6.1 Natural models

- Diseases share similar environmental exposure, genetics and progression patterns.
- Provide insights into spontaneous disease progression/development.
- Capture the complexity of multifactorial diseases (genetic predisposition, epigenetic factors).
- May directly translate to human systems.

### 6.2 Artificial models

- Control over variables- easier to establish causal relationships/reduce variability.
- Reproducibility.
- Enable focused studies on specific pathways/mechanisms that may not be apparent in natural models.
- Scalable.
- Accessible.

In CKD, the use of artificial intelligence has thus far focused mainly on models designed to predict disease detection, progression and evolution. AI approaches in this field appear to be largely derived from previously collected or “historic” data and mathematical modelling techniques (101). For example, in human research, in 2019 a prediction model of DKD progression used natural language processing in hospital settings to extract and identify patient information from electronic health records. Large-scale datasets were analysed using a logistic regression prediction model to determine the long-term DKD outcomes (102). Other studies have used Bayesian mathematical models to characterise and predict CKD progression (103). Another example of AI use is of long short-term memory, a mathematical model based on recurrent neural networks designed to process sequences of data over time through short-term memory mechanisms (104).

In veterinary research, an artificial neural network (ANN), a type of AI model that recognises patterns in data, was used to predict CKD in ageing cats using information collected from veterinary hospitals. The model evaluated a population of cats aged (≥7 years) at risk of developing CKD, showing a sensitivity of 87% and a positive predictive value of 53%, meaning that approximately half of the cats identified as at risk developed CKD within 12 months (105). In dogs, a deep learning model was developed to classify CKD stages from renal ultrasonographic images. Although the model trained successfully and demonstrated improved performance in identifying IRIS CKD Stage 3, its overall precision and accuracy remained low (0.46 and 0.50 respectively) (106).

Therefore, further AI model development and validation studies are underway, although research and clinical applications remain considerably more studied in human medicine than in veterinary medicine.

Insights from human studies and research in domestic animals (Figure 3), combined with technological advances and the development of a more precise biomarker panel, may enhance the accuracy of CKD detection and treatment.

**Figure 3:**
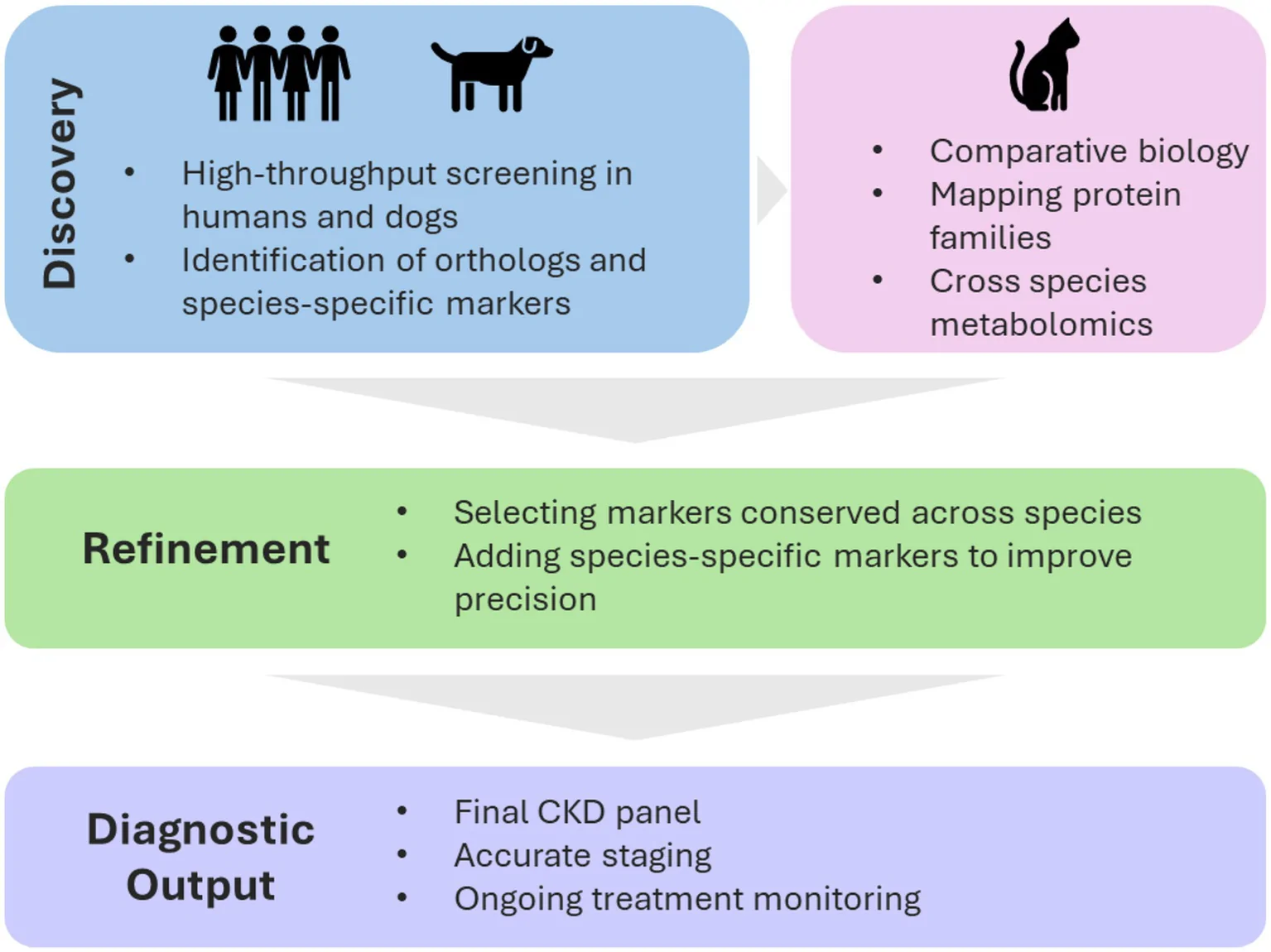
Biomarkers flow for the detection of diseases in veterinary and human medicine. Development of biomarkers identified in dogs and humans may aid in defining a biomarker panel to accurately determine CKD stage. Detection of additional biomarkers in other species may further support treatment improvement by measurement of orthologs (protein markers) or the same metabolites in more than one species with CKD.

## 7 Implications for veterinary medicine

In veterinary medicine, the companion animal (dogs and cats) renal disease market was valued at $US 15.7 billion, with anticipated growth of 5.5% by 2030 (3). There is major unmet need in this area due to the prevalence of the disease. Predicted increases are largely driven by renal complications in pets, exacerbated by greater longevity (2). However, in agreement with previous work (14), and as with any new pharmaceutical drug or vaccine, we recognise that biomarker development is a time-consuming process prior to clinical application in veterinary medicine; nevertheless, such development may be necessary to advance diagnostic and prognostic capabilities in clinical practice. Biopsies obtained from dogs in different regions worldwide report an average median survival time of 608 days to determine CKD (107). However, considerable variability exists between individuals, with some dogs surviving longer than others (107). As performing tissue biopsies is often invasive or impractical, biomarkers may offer a valuable alternative by enabling the prediction of disease progression without the need for invasive procedures such as biopsy.

## 8 Future directions

The understanding of CKD has substantially advanced through biomedical research, and its translation into clinical practice has improved outcomes in humans, for instance, in the detection of kidney disease. However, challenges remain, notably in achieving sufficient specificity in CKD staging and in predicting disease trajectory. The development of biomarkers panels capable to detect progression efficiently and promptly using multidimensional analyses where necessary and in a cost-effective manner has the potential to enable more personalised care in both humans and companion animals. Building on the work described above, the integration of current technologies with artificial intelligence approaches suggest that further investigations in veterinary medicine will yield algorithms and markers for helping patient stratification in CKD. Findings in canine studies maybe relevant to human biomedical research and vice versa. Multiomics approaches focused on the establishment of biomarker panels across CKD stages may subsequently inform treatment strategies and support the development of novel pharmaceutical and other therapeutic interventions for companion animals. Such precision medicine approaches require specific diagnostics, as highlighted throughout this review, and represent a rapidly expanding field that is likely to bring significant changes to clinical decision-making.

## 9 Conclusion

Dogs and cats both develop CKD; however, unlike cats, dogs share several physiological and pathological characteristics with humans. Research in dogs, including senior dogs, therefore provides a valuable model for investigating CKD in humans. Advances in human biomarker research have improved the diagnosis and prognostication of CKD; but there is still a major unmet need in this area. Thus, further work on companion animals and kidney disease can support human health research. MicroRNAs offer some insight into disease stage and underlying molecular pathways, while panels of protein biomarkers can predict kidney failure, progression to ESRD, and mortality. This review has synthesised key findings on microRNAs and protein biomarkers from clinical studies in humans and companion animals. Similarities may support improved diagnosis and prognostication of CKD in companion animals, enhance disease management, and inform veterinary clinical practice.

## 10 Limitations

The main limitation of this review is the scarcity of studies investigating biomarkers in companion animals. In particular, omics-based studies, including proteomic and transcriptomic approaches, are far less common in companion animals than in humans and are lacking across many tissue types. In addition, comparisons of biomarkers must be interpreted with caution because of methodological differences between studies, and should take into account factors such as sample size, sample type, and breed, which are of particular relevance in companion animal research. Furthermore, certain biomarkers may exhibit species-specific expression patterns, limiting direct cross-species comparability.
